# Increasing organ donation by presumed consent and allocation priority: Chile

**DOI:** 10.2471/BLT.14.139535

**Published:** 2015-03-01

**Authors:** Alejandra Zúñiga-Fajuri

**Affiliations:** a University of Valparaíso, Escuela de Derecho, Errázuriz 2^1^20, Valparaíso, 2362736, Chile.

## Abstract

Chile, a middle-income country, recently joined Israel and Singapore as the world’s only countries to require reciprocity as a precondition for organ transplantation. The Chilean reform includes opt-out provisions designed to foster donation and priority for organ transplantation for registered people. Although the reform has had serious difficulties in achieving its mission, it can be reviewed by other countries that seek to address the serious shortage of organs. As increased organ donation can substantially enhance or save more lives, the effect on organ availability due to incentives arising from rules of preference should not be underestimated.

## Résumé

Le Chili, pays à revenu intermédiaire, a récemment rejoint Israël et Singapour dans le groupe des pays du monde à exiger la réciprocité comme condition préalable à la transplantation d'organes. La réforme chilienne contient des dispositions de retrait conçues pour favoriser les dons et donner la priorité des dons d'organes aux personnes inscrites. Bien que la réforme ait eu des difficultés à atteindre son but, elle peut être adoptée par d'autres pays qui cherchent à résoudre l'inquiétante pénurie d'organes. En effet, des dons accrus d'organes peuvent considérablement améliorer ou sauver davantage de vies. L'effet sur la disponibilité des organes, due à des incitations qui découlent des règles de préférence, ne devrait pas être sous-estimé.

## Resumen

Chile, un país de ingresos medios, se ha sumado recientemente a Israel y Singapur como los únicos países del mundo que exigen reciprocidad como condición previa para el trasplante de órganos. La reforma de Chile incluye disposiciones de exclusión voluntaria diseñadas para fomentar la donación y la prioridad del trasplante de órganos para personas registradas. Aunque la reforma ha experimentado dificultades importantes para lograr su misión, otros países que buscan atender la grave escasez de órganos podrían revisarla. Dado que el aumento de la donación de órganos puede mejorar considerablemente o salvar más vidas, cabe no subestimar el efecto sobre la disponibilidad de órganos debido a los incentivos derivados de las reglas de preferencia.

## ملخص

انضمت شيلي، البلد المتوسط الدخل، في الآونة الأخيرة إلى إسرائيل وسنغافورة باعتبارهم البلدان الوحيدة في العالم التي تشترط المعاملة بالمثل كشرط مسبق لزرع الأعضاء. ويشمل الإصلاح في شيلي نصوصاً لاختيار عدم المشاركة تم وضعها لتعزيز التبرع والأولوية لزرع الأعضاء للأشخاص المسجلين. ورغم أن الإصلاح واجه صعوبات كبيرة لتحقيق مهمته، إلا أنه يمكن استعراضه عن طريق البلدان الأخرى التي تسعى إلى معالجة النقص الشديد في الأعضاء. ولأن زيادة التبرع بالأعضاء يمكنها تعزيز أو إنقاذ مزيد من الأرواح بشكل كبير، فلا ينبغي بخس تقدير التأثير على إتاحة الأعضاء بسبب الحوافز الناجمة عن قواعد التفضيل.

## 摘要

智利这个中等收入国家最近与以色列和新加坡走到了一起，成为世界上仅有的几个要求以互惠原则作为器官移植先决条件的国家。智利的改革包括旨在促进为登记人士提供器官移植的捐献和优先级的选择退出条款。虽然实现改革的使命困难重重，但其他寻求解决器官严重短缺问题的国家可以从中得到借鉴评估。因为器官捐赠增加可以大大提高更多人的生命质量，拯救更多的生命，因此不应低估由于偏好规则带来的激励对器官来源的影响。

## Резюме

Чили, страна со средним уровнем доходов, недавно присоединились к Израилю и Сингапуру, которые являются единственными странами мира, требующими взаимности в качестве предварительного условия для трансплантации органов. Чилийская реформа предусматривает принятие положений об отказе, которые призваны способствовать донорству и установлению приоритета при трансплантации органов для зарегистрированных людей. Хотя при достижении своей цели реформа столкнулась с серьезными трудностями, ее стоит изучить другим странам, которые стремятся решить проблему серьезной нехватки органов. Поскольку увеличение объемов донорства органов может существенно продлить или спасти множество жизней, не следует недооценивать влияния мер стимулирования, вытекающих из правил предпочтения, на доступность органов.

## Introduction

In recent years, technical, political and public opinion in many countries has shifted towards the view that opt-out provisions can help promote organ donation. Two components of transplantation legislation – presumed consent and allocation priority – are thought to increase the donor population by decreasing the ease of opting out and giving registered donors priority among the pool of individuals in need of an organ transplant. The joint implementation of these components is believed to have yielded beneficial effects in Israel and Singapore.^1^^,^^2^ To address disappointing results in the number of organ donors, Chile amended its Organ Donor Act in 2013 to include these components.

This paper discusses opting out and prioritizing allocation to increase organ donors in the light of the Chilean experience. Although transplantation legislation in Chile is not ideal, it sets a precedent. The experience gained may be a useful resource to countries seeking to increase their pool of potential organ donors.

## Legislation to increase donations

Organ transplantation statutes can be categorized on the basis of the nature of donor consent, the means of exercising consent and the relationship between consent status and prioritization for transplant receipt. Explicit opt-in organ donation systems require an individual to express their consent to become a potential donor, whereas explicit opt-out systems presume consent unless an individual expresses their refusal to become a potential donor.^3^ Universal donor systems place no special conditions on the relationship between donor status and transplant allocation, whereas contingent entitlement systems mandate reciprocity by giving consenting potential donors priority for transplant receipt.

Explicit opt-out laws have long been among the major interventions used to increase the pool of potential donors in countries such as Austria, Belgium, the Czech Republic, Finland, France, Greece, Hungary, Israel, Italy, Luxembourg, Norway, Poland, Slovenia, Spain, Sweden and Turkey. There is evidence that supports the association between presumed consent and increased donation rates and that countries with opt-out laws have rates 25 to 30% higher than those in countries requiring explicit consent.^4^ However, presumed consent appears to be only one of several influential factors.^5^ Other factors include potential donor availability, transplantation infrastructure, health care spending and public attitudes,^6^ as well as familial consent and donor registries.^7^

In 1987, Singapore passed the Human Organ Transplant Act, which applies the priority rule with an opt-out system.^2^ If a person objects to donating their organs upon death, they give up priority for receiving an organ should they need one in future. The opt-out with priority system provides a dual-incentive for donation: avoiding the cost of opting-out and receiving priority on the waiting list.^8^ A concern with combining the opt-out and priority allocation system is that the priority rule cannot prevent the free-rider problem if the introduction of an opt-out system has already generated a sufficient organ supply.^9^ Singapore’s combination of presumed consent and priority status appears to have been somewhat successful in increasing organ donations.^10^^,^^11^

In January 2010, the Organ Transplant Act 2008 came into effect in Israel, which governs organ donation and allocation. The new law introduced a priority point system to motivate individuals to donate their organs. This system rewards those who are willing to donate an organ with preferential status as a recipient. A person can gain priority points by signing a donor card, making a non-directed/non-specified organ donation during their lifetime, or being a first-degree relative signing a donor card or consenting to procurement of organs after death. The resulting tiered system includes maximum priority, regular priority and second priority. Maximum priority is granted to candidates if: (i) consent has been given for organ donation from a deceased first-degree relative or (ii) they donated a kidney, a lobe of their liver or a lobe of their lungs in the course of their life to a non-specified recipient. Regular priority is given to candidates who hold a donor card, that is, those who have consented to donate their organs after their death. Second priority is granted to candidates with a first-degree relative who holds a donor card, even if they do not hold a donor card themselves. The act has led to a record number of signed donor cards and there has been a significant increase in the numbers of transplants.^1^

## The Chilean experience

In Chile, transplantation expenses are covered by the transplant recipient. For 80% of the population, health coverage is public and free of charge. The remaining 20% hold private health insurance. No person is denied an organ donation on grounds of financial incapacity.

The number of donors in Chile increased from 52 in 1993 to 147 in 2000.^12^ However, the increase halted and after 2006, organ donations started to decrease. In response, Chile introduced the Organ Donor Act, Law 20413 in January 2010, which established a presumed consent system and a transplantation coordinating committee. Additionally, the law required the Office of Vital Records to keep an official non-donor registry comprising all individuals who opted out.

The number of individuals who donated organs reached a 15-year low of 92 in 2010, a decrease of 17% from 2009 and 40% from 2006. The mean donor frequency during 2010–2011 was 5.95 donors per million population, 29% less than the frequency of 8.31 donors per million population observed during 2000–2009.^13^ Even when accounting for the adverse effects of the earthquake that occurred in February 2010 – such as loss of hospital facilities – these data suggest that the decreasing trend first noted in 2007 was exacerbated in 2010 by the new law. 

In December 2011, 2052 adult Chileans had opted out while obtaining or renewing their identity cards or driver’s licences, which corresponds to 37% of all renewals. By July 2012, 2 780 223 had opted out.^14^ However, Chileans may have been misinformed about the implications of the new law. A survey showed that over 70% of respondents were unaware of the scope of the new law and 16% felt that the organ donation and transplantation system was subject to market forces.^15^ In the same survey, 12% of participants believed that access to procured organs was limited to wealthy individuals, whereas 13% feared that health-care professionals would let registered donors die to harvest their organs.^16^ Finally, opting out was relatively easy: individuals merely had to state their choice when obtaining or renewing identity cards or driver’s licences.

To address this large-scale opt-out, Chile amended the Organ Donor Act with Law 20673 in October 2013. The revision required individuals wishing to become non-donors to submit a notarized statement to the non-donor registry. The amended act also asserts that: “All else being equal, those not registered as non-donors will be entitled to priority in allocation of organs for transplantation purposes.” The registry’s role is now twofold. In addition to documenting the wishes of objectors, it provides an additional tool for transplantation physicians to decide who gets priority. As such, provided there is equal need and compatibility, registered non-donors are not prioritized.

The amendments did not revoke choices made by individuals during the previous law when there were no consequences of being a non-donor. As a result, individuals who chose to be non-donors in 2010–2013 also lost priority in the organ transplantation queue.

It is too early to draw any conclusions about the results of the reform – in particular, whether or not the prioritization rule and the difficulties of opting-out will reverse the numbers. However, knowing that many Chileans mistrust the organ donation system,^15^ one can speculate that the drop and subsequent rebound of organ donation rates between 2007 and 2012 could be due to the introduction of a more complicated process for opting-out. If this is the case, moral legitimacy questions of presumed consent legislation arise – i.e. whether it is morally legitimate to compel people into being organ donors and penalize the ones who opted out by denying them priority.

However, the number of organ donations rebounded in 2011 and 2012 with 113 and 149 organs donated, respectively, but dropped to 103 organs donated in 2013. In 2014, the number of organ donations rebounded again to 123.^12^

## Discussion

To promote organ donation, legislating the principle of priority provides a strong incentive by signalling to people that registering as a non-donor decreases their chance of receiving a donated organ when needed. Such legislation is in place in Israel and Singapore. Singapore has experienced an increase in the number of donors after introducing a priority system, although the effect of the priority system is unclear because a presumed consent system was implemented at the same time. Preliminary results in Israel, which does not have a concomitant policy of presumed consent, are promising, showing a significant increase in both deceased and living organ donation.^17^

Legislation of the principle of priority offers a transparent process of prioritizing potential recipients, by serving as a source of external justification. If a person can be an organ recipient, they should also be able to give an organ, and vice versa.^18^ Given that pragmatism prevails in society, it is hoped that the priority rule will prompt people who opted out of donor programmes to reconsider their choice.

The principle is consistent with the view that a fair concept of justice calls for reciprocal altruism, because organs may be considered a scarce societal resource – i.e. the demand for donated organs is higher than the supply of such organs. It also justifies the perceived unfair action of free-riders; those who are willing to receive an organ but unwilling to donate one. The willingness to be a donor in exchange for eligibility to receive an organ seems a basic moral requisite. Further, many people believe that it would be incorrect to allow organ recipients the right to refuse to donate upon their death. Laws can induce desirable cultural changes and help bring about more cohesive, caring, responsible societies.

To some, reciprocity is derived from a more general moral burden called the duty of mutual aid. Many countries use general moral duty as a foundation of laws. This duty places a legal obligation on any citizen – not just medical or law enforcement personnel – who encounters a person in serious danger to assist the individual in a way that does not cause cost or risk to the potential rescuer.^19^

Organs become public goods after being donated for transplantation. The allocation of organs is regulated by the central government in Chile and as for all public goods, everyone in need is entitled to the organs, even free-riders. In Chile, the law sets allocation priorities, not a standard of exclusion. As Jarvis notes: “[t]hose and only those who elect potentially to contribute to the system stand to benefit from it.”^20^ The allocation priorities help select the recipient of the donated organ, if there is more than one matching recipient, by prioritizing those who are on the organ donor list. This will encourage people to stay in the programme and therefore increase the number of potential donors. Also, at a community level there might be a marginal benefit of promoting solidarity and altruism instead of self-interest.

One can speculate that consenting to the postmortem removal of body parts could generate significant costs or risk for the consenters since they might receive suboptimal care if hospitalized in a critical condition. However, hospital staff do not have any financial incentives to notify organ procurement agencies of potential donors under their care. Since presumed consent makes most individuals potential donors, the physicians have no reason to make distinctions between patients.^21^

Another critique against priority incentives is that one should donate organs principally for altruistic reasons and that non-altruistic incentives degrade the altruistic nature of our current system. However, Kolber argues that “priority incentives will not reduce opportunities to act altruistically, because they will increase [the] donors’ range of opportunities. They do not reduce altruistic behaviour, since those with priority are still making a donation; they are just donating to a pool with limited access”.^22^

Although priority rules might reduce altruistic organ donations, this does not mean that reciprocity rules are unfair or arbitrary. Instead, they are designed to prevent those who will not donate from benefiting from those who have agreed to do so. Allocation of scant resources should be decided on the basis of need, yet nothing prevents complementing this rule to promote justice and efficiency. Reciprocity can foster justice in the sense that only those who act with justice will be entitled to justice.^23^ It also fosters efficiency, as negative incentives may help retract choices often made without much thought of the consequences.^24^ As most decisions not to donate tend to be without proper reflection of the consequences, measures – such as priority rules – may encourage solidarity. Because the priority incentives offer donors the possibility of increasing their life expectancy, this provides a strong motivation to donate. With a priority system, people have an incentive to register because they are more likely to gain from the system than to contribute to it.

Policy-makers that are concerned about the shortage of donor organs in their country could study the efforts made by Chile to boost organ donation. Offering registered donors priority for receiving organ transplants may encourage more people to become organ donors. Schemes in which choice is driven by the individual’s interest can also further the community’s interests.
